# The relationship between sensory processing sensitivity and medication sensitivity: brief report

**DOI:** 10.3389/fpsyg.2023.1320695

**Published:** 2024-01-16

**Authors:** Jadzia Jagiellowicz, Bianca P. Acevedo, Teresa Tillmann, Arthur Aron, Elaine N. Aron

**Affiliations:** ^1^Department of Psychology, Stony Brook University, Stony Brook, NY, United States; ^2^Department of Psychological and Brain Sciences, University of California, Santa Barbara, Santa Barbara, CA, United States; ^3^Faculty of Psychology and Educational Sciences, Chair for School and Teacher Research, Ludwig Maximilian University of Munich, Munich, Germany

**Keywords:** medication sensitivity, gender, adverse drug reactions (ADR), sensory processing sensitivity (SPS), pharmacogenomics, personalized and precision medicine (PPM), environmental sensitivity, medication adherence

## Abstract

Sensory processing sensitivity (SPS) is a biological/temperament trait that is associated with greater awareness of and reactivity to the environment, which results in amplified responses to various stimuli, and possibly medications. We investigated the relationship between SPS and medication sensitivity in three studies. Participants (ages 18–81) were recruited from university (Study 1: *N* = 125; Study 2: *N* = 214) and online (Study 3: *N* = 351) samples. In each study, participants completed a medication sensitivity scale, the standard highly sensitive person (HSP) scale to assess SPS, and a negative affectivity (NA) scale as a control variable. All three studies found moderate, significant correlations between SPS and medication sensitivity (*r* = 0.34, *p* < 0.001: *r* = 0.21, *p* = 0.003; *r* = 0.36, *p* < 0.001, respectively). Correlations remained significant, and similar, when controlling for NA and gender; and there were no significant interactions with gender. In sum, our results suggest that SPS is associated with medication sensitivity, even when considering NA and gender. Thus, future work might consider SPS when investigating recommended medication, medication dosage, effectiveness, and adverse drug reactions.

## Introduction

It is clear that when examining response to prescription medications, that many variables may interact to produce positive results or adverse drug reactions (ADRs), such as age, weight, lifestyle, co-morbidities, and genetic variation (Zhou et al., 2015; Haga, 2017). Precision medicine is a field that takes these various factors into account to create personalized medicine tailored to individuals’ lifestyle, genes, and other variables. Coinciding with this goal, researchers have started to examine how traits (particularly those with a biological basis) impact individual differences in drug response (Costello et al., 2014).

Gender has also been investigated in the context of physical sensitivity, including sensitivity to medication and to the perception of physical pain, with some research suggesting that women experience more ADRs (Kando et al., 1995) and pain (Engel-Yeger and Dunn, 2011), compared with men. Collectively, the results of these studies highlight how biologically-based traits and gender may be differentially associated with responsivity and sensitivity to medications.

Individuals who demonstrate heightened sensitivity to medications, based on genetic variations or metabolic differences can experience more ADRs or side effects (Evans and Johnson, 2001; Shuldiner et al., 2009; Kalichman et al., 2022) which can influence healthcare usage (Macy and Ho, 2012; Baliatsas et al., 2015), health information seeking (Faasse et al., 2015), as well as treatment adherence, treatment efficacy, and outcomes (Kalichman et al., 2022.) To illustrate, in American patients receiving HIV care, perceived sensitivity to medications was associated with greater experience of antiretroviral side-effects and less compliance with their treatment regimen, which was then associated with increased HIV viral load (Kalichman et al., 2022). Patients with medication sensitivity also needed dosage adjustments or alternative medications for better management (Evans and Johnson, 2001; Shuldiner et al., 2009). For example, differences in drug metabolism and receptor sensitivity linked to the CYP2C19 gene were associated with decreased activation of the anti-blood-clotting drug clopidogrel (Shuldiner et al., 2009). These individuals had to be prescribed an alternative drug or risk a possibly fatal ischemic event. Additionally, individuals with a specific polymorphism in the enzyme thiopurine S-methyltransferase were unable to metabolize various types of thiopurine medications used in leukemia therapy (Evans and Johnson, 2001). These individuals could tolerate only 5–10% of the conventional dose of these medications before developing toxicity.

With the recent advent of pharmacogenetics, researchers have been able to quickly identify genetic polymorphisms associated with individual differences in medication sensitivity (see reviews by Evans and Johnson, 2001; Singh, 2023). Pharmacogenomics is based on the premise that polymorphisms, i.e., differences in the structure of a specific gene or networks of genes, can determine individual differences related to drug metabolism and response. Nevertheless, there are issues with healthcare inequalities due to the cost, the underrepresentation of certain groups in genetic databases, as well as issues with data privacy, informed permission and discrimination based on genetic analyses (see review by Singh, 2023).

The temperament trait of sensory processing sensitivity (SPS), which is characterized by greater awareness of, and reactivity to, environmental stimuli, both for better and for worse (for reviews, see Aron et al., 2012; Greven et al., 2019), is potentially a very relevant model for understanding medication sensitivity within the broader population. The trait shows fundamental neural differences between individuals in a species, suggesting it could serve as a key biomarker for sensitivity to medication. Since it is found in 20 to 33% of the general population (Lionetti et al., 2019), it could be used to screen a significant percentage of hypersensitivity. In addition to SPS, individuals with various neurologic issues (such as traumatic brain injury and autism) and with chronic pain also experience sensory hypersensitivity (e.g., López-Solà et al., 2014; Callahan and Lim, 2018). About 18% of patients with brain injury report experiencing sensory hypersensitivity subsequent to their injury (Chung and Song, 2016). As such, a review of studies examining patients with post-stroke subjective sensory hypersensitivity (Thielen et al., 2023) found a link between sensory hypersensitivity and lesions to the insula—a brain region commonly shown in fMRI studies of SPS (e.g., Acevedo et al., 2018). Thus, the relationship between SPS and medication sensitivity can serve as an easily measurable marker for sensory hypersensitivity in general.

Also, SPS is expressed differently according to environmental conditions, such that in stressful or chaotic contexts those with high levels of SPS may experience negative arousal, stress, anxiety, and negative affect (for review see Greven et al., 2019). On the other hand, in response to positive stimuli and environments, those with high levels of SPS show larger positive responses than those with low levels. There is evidence, for instance, in investigations exploring brain activity linked to reward mechanisms and the outcomes of interventions for depression (Acevedo et al., 2014; Pluess and Boniwell, 2015). Such research has associated higher levels of SPS with stronger positive reactions, thereby suggesting that tailoring treatments according to sensitivity levels could disproportionately influence the efficacy of treatment outcomes.

Individual differences in SPS—which are associated with stronger response to stimuli, including emotional images, others’ moods, sounds, smells, strong lights and caffeine—may also inform dose-response variations, which are impacted by a variety of factors including environmental influences and underlying physiology (e.g., Allen et al., 2020). For example, one study examining response to evening light and melatonin suppression found that sensitivity was associated with differential responses to varying levels of evening light (Phillips et al., 2019). Specifically, the most sensitive individuals in the study by Phillips et al. (2019) showed melatonin suppression at dim light levels, but the least sensitive participants showed the same level of suppression only when exposed to bright indoor light. Thus, highly sensitive individuals may require lower medication doses.

In addition, consistent with gender differences in medication response, at least one study found significant interaction effects between SPS, gender and self-reported physical symptoms—including back pain, diarrhea, and sore throat (Benham, 2006).

The present study aimed to fill a gap in research by examining the association between medication sensitivity and SPS. We predicted that SPS would be positively associated with sensitivity to medication. In addition, we also controlled for negative affectivity (NA) in each of the three studies because the widely used measure of SPS—the highly sensitive person (HSP) scale—tends to include many negatively-worded items. Thus, we controlled for NA, and examined whether any correlation of medication sensitivity with SPS might be due to NA. Also, given the study showing a greater number of ADRs among women versus men (Kando et al., 1995), we also explored whether gender might contribute a moderating role to the association between SPS and medication sensitivity.

In the three studies described herein, we asked participants recruited from university samples and online to complete self-report measures of SPS (measured with the HSP scale), NA (as a control variable), and medication sensitivity. We predicted that SPS would be positively associated with medication sensitivity, and that gender would interact with SPS, resulting in particularly higher sensitivity to mediations among women high in SPS.

## Materials and methods

### Participants

#### Study 1

Participants of 18+ years who were able to complete an English survey were selected from undergraduate students participating in psychology research. After excluding a participant who did not complete the medication questionnaire, the Study 1 sample was composed of 125 participants (70.40% women; 58% aged 20 to 24; overall range, based on age range choices, was 35% “15–19,” 4% “25–29,” and 2.4% “over 30”). Participants were recruited from a northeastern US university as part of standard mass testing. The study was administered in return for course credit. All participants provided informed consent in accordance with Stony Brook University’s IRB procedures.

#### Study 2

The Study 2 sample consisted of 214 participants (61% women), ages 18 to 77 (*M* = 30.3, SD = 11.8). Approximately 30% of respondents were students and 44% were currently employed. The majority of the sample was college educated: 38% had some college education, 16% had an Associate’s degree, 18% a Bachelor’s degree, 13% a Master’s degree, 1% a professional degree, and 2% a Doctorate; and about 11% of had only completed high school. Our sample was diverse, including 27% Black/African-Americans, 12% Latino/Hispanics, 39% Caucasians, 7% Asians, 6% native Americans, and 5% replied “Other.” Participants were recruited via advertisements, flyers, listservs, social media, and by the University of California, Santa Barbara’s (UCSB) Subject Pool to participate in a 30-min online survey on “Personality, Behavior, and Emotions.” For participating in the study, subjects were entered into a prize drawing for a $25 gift card. All participants provided informed consent in accordance with UCSB’s IRB procedures.

#### Study 3

Individuals aged 18 years or more were invited to participate. A sufficient knowledge of the English language was necessary. This was confirmed by participation in the study. Participants (*N* = 351; 45% women; *M* age = 35.96, SD = 10.80, range 19 to 81) were recruited online using the crowdsourcing marketplace Amazon Mechanical Turk. Our sample included 6.60% participants of Eastern/Asian, 9.40% Black/African-American, 6.80% Latino/Hispanic/Spanish and 76.90% White/European ancestry. Two participants assigned themselves to the category “Native American,” two additional participants chose the option “other.” For *n* = 349 of the final sample, English was the native language. Nine of the Study 3 participants were excluded from this data set (see “Data analysis” section). The subjects were asked to answer some “Personality” questions. Participants were not remunerated by UCSB, although they were remunerated by Mechanical Turk. All participants provided informed consent in accordance with the University of California–Berkeley Institutional Review Board (IRB).

### Measures

#### Sensory-processing sensitivity (SPS)

Sensory processing sensitivity (SPS) was measured with the standard highly sensitive person (HSP) scale (Aron and Aron, 1997). The HSP scale includes 27 items measuring characteristics such as awareness to subtleties in the environment; sensitivity to scents, noise, lights, fabrics, pain, and caffeine; and the tendency to become overwhelmed in the presence of many stimuli. The HSP scale has been shown to represent a valid and reliable measure of the trait (for a review, see Greven et al., 2019) All items are answered on a 7-step Likert-scale. Reliability measures for each study are as follows: Study 1 (α = 0.88), Study 2 (α = 0.93), and Study 3 (α = 0.93).

#### Negative affectivity

Previous studies of SPS (e.g., Aron and Aron, 1997; Acevedo et al., 2014; Lionetti et al., 2019) have typically controlled for NA, due to the HSP scale’s moderate to high correlation with negative affect (approximately 0.3). Studies 1 and 3 measured NA with two commonly used items asking, “Are you prone to fears?” and “Are you prone to depression?” Reliability was moderate (Study 1, α = 0.69) to good (Study 3, α = 0.80) for two of the three studies. As a check, we also conducted all analyses using the two NA items individually, and found nearly identical results.

In Study 2, NA was measured with the two-item Emotional Stability subscale of the Gosling et al. (2003) Ten-Item Personality Inventory (TIPI). The alpha for NA in Study 2 was low (α = 0.46), thus we conducted analyses with both the two-item measure and each item separately.

#### Sensitivity to medication

In Study 1, we used the 7-item medication sensitivity scale (Cohen, 1999), which assesses patient variability with respect to adverse drug reactions using primarily binary responses. In Study 1, only six items of the scale were used and they were slightly adjusted. In particular, open-ended questions of the original scale were re-phrased to elicit binary responses (e.g., “How are you affected by alcohol?” was changed to “Are you sensitive to alcohol?”). Wording was also changed for one item (i.e., “Have you ever had a reaction to epinephrine?” became “Are you anxious after epinephrine?”). Further, questions 1 (“Are you sensitive to any prescription or nonprescription drugs?”) and 7 (“Overall, how would you describe yourself with regard to medication?”) were combined into one general question (i.e., “Are you very sensitive to medicine?”). The reliability of the scale was moderately low (α = 0.55).

Thus, we also ran all analyses using only two general items (“Have you had any side effects from any other prescription or non-prescription drugs?” and “Are you very sensitive to medication?,” α = 0.51), which were nearly identical to items of the validated perceived sensitivity to medicine (PSM) scale (Horne et al., 2013) used in Studies 2 and 3.

The PMS is a 5-item Likert-scale ranging from: “strongly disagree” to 5 = “strongly agree.” Sample items include, “My body is sensitive to medicines” and “I usually have strong reactions to medicines than most people.” Reliability of the PMS scale was strong in Study 2 (α = 0.92) and Study 3 (α = 0.93), and replicates previous findings (see Horne et al., 2013).

### Data analysis

See Table 1 for descriptive statistics of the study variables.

**TABLE 1:** Descriptive statistics of major study variables overall and by gender.

	Study 1		Study 2		Study 3	
	***M***	**SD**	***M***	**SD**	***M***	**SD**
SPS	4.30	0.82	4.64	0.82	3.95	1.06
Females	4.38	0.82	4.69	0.88	4.29	1.05
Males	4.09	0.81	4.53	0.70	3.65	0.97
Medication sensitivity	0.39	0.24	3.12	1.50	2.23	1.02
Females	0.41	0.27	2.94	1.60	2.48	1.16
Males	0.34	0.27	3.43	1.26	2.03	0.84
Negative affectivity	3.67	1.53	4.00	1.28	3.53	1.89
Females	3.68	1.45	4.02	1.33	3.85	1.99
Males	3.64	1.74	3.95	1.16	3.24	1.76

We examined the association between SPS and medication sensitivity with a series of correlations and partial correlations, using version SPSS 16.0 (Study 1) and 22.0 (Studies 2 and 3) of IBM SPSS (IBM Corporation, Armonk, NY, USA). We also conducted partial correlations for SPS and medication sensitivity, controlling for age and gender, as reported in the Results. We used correlations since this was an observational, not experimental design.

For Study 1, data screening included identifying individuals who did not complete scale items. Only one individual had not completed any of the medication sensitivity items. This participant was excluded from the *N* = 126 dataset, leaving 125 participants. One participant had failed to answer one item and another participant had failed to answer two items. The variable mean was calculated from the items that were answered, and the individuals were retained in the data set.

For Study 2, data screening required identifying careless or unmotivated responses prior to performing data analyses due to the nature of online surveys (Meade and Craig, 2012; Dunn et al., 2018). First, we included a direct assessment of careless responses in our survey, where participants were asked three self-report questions: (a) I enjoyed participating in this survey, (b) I worked to the best of my ability on this survey, and (c) I gave this survey my full attention (rated on a 7-point Likert scale). We excluded all cases that were two standard deviations below the mean of the three attention-check items (*M* = 5.58, SD = 1.22). Second, following procedures outlined by Dunn et al. (2018), for each respondent we calculated the intra-individual variability (IRV) index across all items. Respondents with extremely low IRV values across different constructs were excluded from analyses. Third, we excluded respondents that took 10 min or less to complete the survey, as our piloting suggested that the average time to complete the survey was about 25-min. A total of 118 participants were excluded from the original sample of 332, resulting in the final sample of 214 participants.

In Study 3, we included three distractor items that asked participants to answer with a specific response option. Participants who failed these items (*n* = 3) were taken out of the data set. Furthermore, the data set was checked for unusual patterns, such as the use of one answering option across a whole scale (with focus on the HSP scale). Based on the observed patterns, 5 additional participants were taken out of the data set. Furthermore, one participant was taken out based on the fact that his mean (*M* = 1.83) on one of the scales was significantly lower than those of the general data set (i.e., three standard deviations below the mean, *M* = 4.61, SD = 0.85).

## Results

### Study 1

The correlation between SPS and the modified medication sensitivity measure by Cohen (1999) was *r* = 0.34, *p* < 0.001 (and with just the two general medication sensitivity items, *r* = 0.31, *p* = 0.001). The correlation of the modified medication sensitivity measure with SPS, after controlling for NA was *rp* = 0.30, *p* = 0.001. Controlling for gender had a minimal effect on the correlation of medication sensitivity with SPS, *rp* = 0.33, *p* < 0.001; and also, when controlling for gender and NA, *rp* = 0.28, *p* = 0.002. Partial correlations of SPS with the general two-item medication sensitivity measure were similar to those with the full measure when controlling for NA, *rp* = 0.29, *p* = 0.001; and when controlling for gender and NA, *rp* = 0.31, *p* < 0.001. There was no significant interaction of gender and SPS with either the full scale (ßinteraction = −0.25; *p* = 0.677), or the 2-item medication sensitivity measure (ß interaction = −0.74; *p* = 0.222). In sum, Study 1 showed a moderate and significant association of SPS with medication sensitivity, whether or not controlling for NA and gender, and there was no interaction with gender.

### Study 2

Sensory processing sensitivity (SPS) was significantly correlated with medication sensitivity (*r* = 0.21, *p* = 0.003), including when controlling for NA (*rp* = 0.22, *p* = 0.001); and when controlling for the two NA items individually (*rp* = 0.24, *p* = 0.001; *rp* = 0.21, *p* = 0.003). As in Study 1, the interaction of gender and SPS with medication sensitivity was not significant (ßinteraction = −1.83, *p* = 0.069), and controlling for gender had a very minor effect on the correlation between SPS and medication sensitivity (*rp* = 0.22, *p* = 0.002). Also, controlling for NA and the individual NA items had little impact on the association between SPS and medication sensitivity (*rp* = 0.23, *p* < 0.001; *rp* = 0.21, *p* < 0.001; *rp* = 0.22, *p* = 0.002). In sum, consistent with Study 1 results, Study 2 found a small to moderate, but significant, association between SPS and medication sensitivity.

### Study 3

Study 3 showed that SPS was positively associated with medication sensitivity (*r* = 0.36, *p* < 0.001), even when controlling for NA (*rp* = 0.27, *p* < 0.001) replicating results from Studies 1 and 2. Also, as in Studies 1 and 2, the interaction of gender and SPS with sensitivity to medicine was not significant (ßinteraction = −0.23, *p* = 0.411); and controlling for gender had a very minor effect on the correlation between SPS and medication sensitivity (*rp* = 0.32, *p* < 0.001). Controlling for gender and NA (*rp* = 0.23, *p* < 0.001) resulted in only a small decrease in results. In sum, Study 3 replicated Studies 1 and 2, but with a larger sample. As in Studies 1 and 2, Study 3 showed a moderate and significant association between SPS and medication sensitivity, but did not find a significant interaction with gender.

## Discussion

Previous studies have shown that sensory processing sensitivity (SPS) is associated with stronger effects in response to stimuli, both for better and for worse (for review see Jagiellowicz et al., 2020). Also, some studies have shown that individual differences in genetics, biologically-based traits and gender may predict medication sensitivity (Costello et al., 2014). Thus, the set of three studies reported herein examined, for the first time, both the unique association between SPS and medication sensitivity, as well as the association when accounting for gender and negative affect. Results showed a significant correlation between SPS and medication sensitivity, in line with research and theory showing that SPS is associated with more intense reactions to various stimuli (Wachs, 2013; Acevedo et al., 2014; Jagiellowicz et al., 2016; Aron et al., 2019). These results suggest that the physical manifestations of a lower threshold to ADRs would be indistinguishable from a more intense reaction to the same level of stimuli. Thus, we propose that, instead of assuming that ADRs to medications are entirely psychogenic (Ong et al., 2004), that they may be partly due to the more responsive physiology found in some individuals, such as those with the biologically-based temperament trait of SPS (Acevedo et al., 2014; Jagiellowicz et al., 2016; Aron et al., 2019; Acevedo, 2020).

### Limitations and strengths

The present set of studies are exploratory, and as such, are not without limitations. Notably, we used different measures of medication sensitivity in Study 1 versus Studies 2 and 3. Study 1 adapted a medication sensitivity scale. However, results were replicated in Studies 2 and 3 which used a well-validated measure of medication sensitivity. Also, Studies 2 and 3 recruited broader samples—differing in age, and gender distribution—thus, increasing the generalizability of the pattern of results.

Also, it is possible that there may have been some response bias in the present research. Thus, future studies may implement more objective measures of medication sensitivity to address this limitation. For example, future studies would benefit from objective measures of medication sensitivity, such as physician reports or physiological measures; as well as randomized control studies. Also, forthcoming work may consider pre-existing conditions, diagnoses, and the medication status of the respondents.

Nevertheless, these studies provide a foundation for future research by showing that there is a significant relationship between SPS and medication sensitivity, even when controlling for NA. For example, while some studies have proposed that ADRs are mostly psychogenic (Ong et al., 2004), the present findings with respect to SPS suggest that they are not necessarily due to negative associations or trauma. Specifically, we found a nearly identical pattern of results when controlling for NA. In addition, our lack of significant gender differences for medication sensitivity among those with high SPS was consistent with some of the literature (Magharious et al., 1998; Applebaum et al., 2009). Thus, the results reported herein suggest that medication sensitivity observed among those with high SPS may be due to a biological sensitivity to environmental influences inherent in the SPS trait. The HSP scale is also easy to administer, and less costly than pharmacogenetic approaches, thus ameliorating some of the accessibility issues associated with these methodologies. As such, these findings suggest that SPS might be important to consider in precision (personalized) medicine, when deciding medication dosage, and when screening patients that may be susceptible to ADRs.

## Conclusion

In sum, results from the three studies reported on herein suggest that SPS may be associated with a higher sensitivity to medications. Thus, SPS might be important to investigate in future research and practice when considering medication dosage, medication effectiveness, and adverse drug reactions.

In addition, this work might have implications for personalized medicine, since the relationship between SPS and medication sensitivity can serve as an easily measurable marker for sensory hypersensitivity in general.

## Data availability statement

The original contributions presented in this study are included in this article/supplementary materials, further inquiries can be directed to arthur.aron@stonybrook.edu.

## Ethics statement

The studies involving humans were approved by Institutional Review Board (IRB), Stony Brook University (Study 1); Institutional Review Board (IRB), University of California, Santa Barbara (Study 2); Institutional Review Board (IRB), University of California, Berkeley (Study 3). The studies were conducted in accordance with the local legislation and institutional requirements. The participants provided their written informed consent to participate in this study.

## Author contributions

JJ: Conceptualization, Writing – original draft, Project administration, Formal analysis, Investigation, Methodology, Writing – review and editing. AA: Data curation, Supervision, Writing – review and editing. EA: Supervision, Writing – review and editing. BA: Project administration, Formal analysis, Investigation, Methodology, Writing – original draft, Writing – review and editing. TT: Project administration, Formal analysis, Investigation, Methodology, Writing – original draft, Writing – review and editing.
